# Impacto de un programa de prevención indicada en adolescentes con comportamientos de riesgo

**DOI:** 10.23938/ASSN.1173

**Published:** 2026-08-19

**Authors:** Garikoitz Mendigutxia, José J. López-Goñi

**Affiliations:** 1 Programa Suspertu Fundación Proyecto Hombre de Navarra Pamplona España; 2 Universidad Pública de Navarra Departamento de Ciencias de la Salud Pamplona España; 3 Instituto de Investigación Sanitaria de Navarra (IdiSNA) España

**Keywords:** Adolescente, Conducta de Riesgo, Abuso de Cannabis, Servicios Preventivos de Salud, Trastornos Mentales, Adolescent, Risk-Taking, Cannabis Use Disorder, Preventive Health Services, Mental Disorders

## Abstract

**Fundamento:**

La prevención indicada dirigida a adolescentes en situación de riesgo psicosocial representa una estrategia prioritaria en salud pública, aunque la evidencia empírica sobre su eficacia es limitada en el contexto español. El objetivo es evaluar el impacto de un programa de prevención sobre variables de personalidad, competencias personales, sintomatología psicopatológica, consumo de cannabis y otras conductas de riesgo.

**Metodología:**

Diseño cuasi-experimental con medidas en preintervención, posintervención y seguimiento a los tres meses de adolescentes atendidos en el Programa Suspertu de la Fundación Proyecto Hombre de Navarra entre marzo de 2014 y junio de 2017. Se administraron instrumentos validados de personalidad, psicopatología y conductas de riesgo. Se realizaron análisis de medidas repetidas, con cálculo del tamaño del efecto mediante *d* de Cohen.

**Resultados:**

Se incluyeron 61 adolescentes (44 hombres; 72,1%) con edad media 16,7 años (DT=2,0). Se observaron cambios estadísticamente significativos en variables de personalidad, mejorías en competencias personales (autoestima, autoeficacia, planificación, toma de decisiones) y en todas las dimensiones del Listado de Síntomas-90-Revisado, tanto al finalizar la intervención como en el seguimiento. El consumo diario de cannabis se redujo del 45,5% al 4,5% en hombres y del 37,5% al 18,8% en mujeres. Se registró un descenso generalizado en otras conductas de riesgo.

**Conclusiones:**

Los resultados respaldan la eficacia del Programa Suspertu como intervención de prevención indicada, con efectos positivos mantenidos a los tres meses de seguimiento. Se subraya la importancia de considerar el sexo como variable diferenciadora en el diseño y la implementación de este tipo de programas.

## Abstract (en)

**Background:**

Indicated prevention targeting adolescents at psychosocial risk is a public health priority, yet evidence on its effectiveness in Spain remains limited. This study evaluates the impact of a preventive program on personality traits, personal competencies, psychopathological symptoms, cannabis use, and other risk behaviors.

**Methods:**

A quasi-experimental design with three assessment points (pre-intervention, post-intervention, and three-month follow-up) was used. Participants were adolescents recruited though the Suspertu Programme of *Fundación Proyecto Hombre Navarra* between March 2014 and June 2017. Validated instruments assessing personality, psychopathology, and risk behaviors were administered. Statistical analyses included repeated-measures general linear models, effect sizes were estimated using Cohen’s *d*.

**Results:**

The sample comprised 61 adolescents (44 males, 72.1%), with a mean age of 16.7 years (SD = 2.0). Statistically significant changes were observed in personality variables and improvements in personal competencies, including self-esteem, self-efficacy, planning, and decision-making. Significant improvements were also observed across all Symptom Checklist-90-Revised dimensions at both post-intervention and follow-up. Daily cannabis use decreased from 45.5% to 4.5% among males and from 37.5% to 18.8% among females. A general reduction in other risk behaviors was also observed.

**Conclusions:**

These findings support the effectiveness of the Suspertu Programme as an indicated prevention intervention, with positive effects maintained at three-month follow-up. The findings also underscore the importance of considering sex as a potential differentiating factor when designing and implementation indicated prevention programs.

## INTRODUCCIÓN

La adolescencia es una etapa especialmente vulnerable a la aparición de conductas de riesgo que comprometen la salud física, psicológica y social. Entre ellas destacan el consumo precoz de sustancias psicoactivas, la impulsividad, la conducta antisocial o el bajo rendimiento escolar, con una edad de inicio progresivamente más temprana y patrones que muestran una tendencia hacia la normalización entre la población adolescente^1^. En España, la Encuesta sobre Uso de Drogas en Enseñanzas Secundarias (ESTUDES) 2025 señala que el 21,0% de los adolescentes entre 14 y 18 años ha consumido cannabis alguna vez en la vida y el 11,6% en el último mes, con una edad media de inicio de 14,8 años^2^. Navarra se sitúa entre las comunidades con mayor prevalencia nacional en consumo de alcohol (77,8%) y presenta tasas superiores a la media española en consumo de cannabis, lo que refuerza la necesidad de desarrollar estrategias preventivas eficaces dirigidas a esta población^2^. Para orientar las intervenciones preventivas, es fundamental identificar los factores psicológicos asociados a los comportamientos de riesgo.

Diversas variables psicológicas actúan como predictores consistentes de estos comportamientos. La búsqueda de sensaciones se asocia directamente con el inicio temprano del consumo de sustancias^3^ y, aunque parcialmente adaptativa en la adolescencia, amplifica el riesgo cuando no se acompaña de habilidades de autorregulación^4^. La autoestima, la autoeficacia y la resiliencia se relacionan entre sí en la adolescencia y se consideran factores protectores frente al riesgo psicosocial^5^. Asimismo, los adolescentes con consumo problemático presentan mayores niveles de sintomatología psicopatológica, como ansiedad, depresión y hostilidad, y el cannabis puede utilizarse como estrategia de regulación emocional^6^^,^^7^.

Las intervenciones de prevención indicada, centradas en adolescentes que presentan signos de riesgo, permiten realizar una adaptación más específica a las necesidades individuales e influir en variables mediadoras clave^8^. Sin embargo, en el contexto español son infrecuentes los estudios que evalúen su eficacia en entornos comunitarios reales con rigor metodológico^9^. El Programa Suspertu, desarrollado por la Fundación Proyecto Hombre de Navarra desde 1997, es una intervención cognitivo-conductual consolidada con evidencia empírica previa que trabaja competencias personales y sociales para reducir el consumo de sustancias y mejorar el ajuste psicológico en adolescentes y sus familias. En concreto, se ha encontrado que la intervención con las familias muestra cambios positivos en la vivencia de la parentalidad y de la convivencia^10^, y que las familias que logran más altas terapéuticas son aquellas que acuden con sus hijos o hijas^11^. La aportación específica de este estudio es evaluar el efecto de la intervención en los propios adolescentes de ambos sexos.

El presente estudio tiene como objetivo evaluar la eficacia del Programa Suspertu en variables de personalidad y sintomatología psicopatológica asociadas al consumo de cannabis y otras conductas de riesgo en adolescentes, analizando su evolución desde el inicio hasta la finalización de la intervención y a los tres meses de seguimiento, con atención a las diferencias en función del sexo.

## MATERIAL Y MÉTODOS

### Diseño y participantes

Se llevó a cabo un estudio cuasi-experimental con adolescentes de entre 13 y 20 años con conductas de riesgo psicosocial (consumo de sustancias, conducta antisocial o bajo rendimiento escolar) atendidos en el Programa Suspertu de la Fundación Proyecto Hombre de Navarra, con sede en Pamplona (España) y cobertura para toda la Comunidad Foral de Navarra. El periodo de reclutamiento consecutivo se extendió desde marzo de 2014 hasta junio de 2017.

Los criterios de inclusión fueron: 1) firmar el consentimiento informado (en el caso de jóvenes menores de edad, también lo firmaron los progenitores o tutores legales), 2) estar acompañados de sus familias o referentes en caso de proceder de un recurso de protección, y 3) haber cumplimentado todos los instrumentos de evaluación.

La investigación contó con la autorización del Comité de Ética, Experimentación Animal y Bioseguridad de la Universidad Pública de Navarra (PI-003/14).

### Intervención

El Programa Suspertu es una intervención de prevención indicada de orientación cognitivo-conductual fundamentada en los modelos teóricos relacionados con los factores de riesgo y de protección. Está dirigida a adolescentes con conductas de riesgo y a sus familias.

La intervención sobre el adolescente es de carácter individual, y no se realiza trabajo en grupos. Con una frecuencia semanal, se prolonga durante aproximadamente 6 meses. A partir de la evaluación inicial estandarizada para la detección de los principales factores de riesgo y de protección, se diseña un plan de trabajo personalizado estructurado en módulos que abordan competencias personales y sociales (autoestima, autoeficacia, habilidades sociales, planificación y toma de decisiones, regulación emocional y prevención de recaídas en el consumo de sustancias).

De forma paralela, los progenitores o figuras de cuidado reciben una intervención familiar específica orientada a reforzar las pautas educativas, mejorar la comunicación parento-filial y favorecer un entorno protector. Además de las sesiones individuales semanales con cada familia, los progenitores también acuden con frecuencia semanal a una *Escuela de padres y madres*, dividida en dos módulos de seis sesiones cada uno. Tal y como ya se ha señalado, la intervención familiar fue evaluada en estudios previos^10^^,^^11^.

La intervención es desarrollada por un equipo multidisciplinar (profesionales de psicología, educación y trabajo social) con formación específica de máster, cuatro años de experiencia mínima y supervisión clínica continuada por parte del propio equipo.

Tras la finalización de la intervención se establece un seguimiento sistemático a los tres meses, en el que se administra de nuevo el protocolo de evaluación y se valora el mantenimiento de los cambios alcanzados.

### Procedimiento de evaluación

En la fase de acogida se solicitó el consentimiento informado del adolescente y, en su caso, de los progenitores o tutores legales.

La recogida de datos preintervención se realizó mediante tres o cuatro entrevistas individuales en el contexto habitual de la valoración inicial del programa, integrando la información clínica y la propia de la investigación.

Una vez concluida la intervención individualizada, se administró nuevamente el protocolo (datos posintervención). Finalmente, a los tres meses de la finalización, los participantes acudieron a una nueva sesión de evaluación para valorar el mantenimiento de los cambios (datos de seguimiento). En las tres ocasiones se administraron los mismos instrumentos en condiciones equivalentes.

### Variables e instrumentos

En la valoración inicial del programa se recogieron distintas variables sociodemográficas:

- sexo (hombre, mujer),
- edad (años),
- núcleo de convivencia (junto a padres y hermanos, padres separados/divorciados, solo con mi madre, con mi madre y su nueva pareja, solo con mi padre, con familia adoptiva),
- nivel de estudios (enseñanza secundaria obligatoria/ESO, bachiller, formación profesional/FP, universidad),
- antecedentes judiciales (sí, no),
- diagnóstico psiquiátrico previo (sí, no).

En los tres momentos de valoración se administró el mismo protocolo de evaluación con los siguientes instrumentos:

- *Substance Use Risk Profile Scale* (SURPS)^12^. Evalúa cuatro factores de personalidad mediante 23 ítems (escala Likert de 1 a 4) agrupados en cuatro subescalas: Sensibilidad a la Ansiedad (5 ítems), Introversión/Desamparo (7 ítems), Búsqueda de Sensaciones (6 ítems) e Impulsividad (5 ítems).
- *Escala de Autoeficacia Generalizada*^13^^,^^14^. Escala unidimensional de 10 ítems tipo Likert (rango 1-4) que evalúa la percepción de competencia personal ante dificultades. Alfa de Cronbach = 0,82.
- *Escala de Autoestima de Rosenberg*^15^. Escala unidimensional de 10 ítems tipo Likert (rango 1-4) que mide la autoestima global. Alfa de Cronbach = 0,92.
- *Escala de Tolerancia a la Frustración*^16^. Subescala adaptada del *Emotional Quotient Inventory: Youth Version* (EQ-i:YV) que evalúa la capacidad para resistir situaciones adversas e impulsos. Ocho ítems tipo Likert (rango 1-5). Alfa de Cronbach = 0,77.
- *Escala de Planificación y Toma de Decisiones*^17^. Adaptación de la *Life Skills Development Scale, Form B* (LSDS-B) que evalúa la percepción de la propia habilidad para planificar y tomar decisiones. 8 ítems tipo Likert (rango 1-7). Alfa de Cronbach = 0,89.
- *Escala de Habilidades Sociales*^18^. Cuestionario de 12 ítems (rango 1-7) con tres dimensiones: habilidades comunicativas, asertividad y resolución de conflictos. Alfa de Cronbach = 0,69.
- *Listado de Síntomas-90-Revisado* (SCL-90-R)^19^. Cuestionario autoadministrado de 90 ítems (escala Likert 0-4) con nueve dimensiones de síntomas primarios (somatización, obsesión-compulsión, sensibilidad interpersonal, depresión, ansiedad, hostilidad, ansiedad fóbica, ideación paranoide y psicoticismo) y tres índices globales: Índice Global de Gravedad (GSI), Total de Síntomas Positivos (PST) e Índice de Distrés de Síntomas Positivos (PSDI). Las puntuaciones se transformaron en percentiles a partir de los baremos de referencia.
- *Communities That Care Youth Survey* (CTCYS)^20^^,^^21^. Cuestionario que recoge el patrón de consumo de cannabis (categorizado en: sin consumo, esporádico, habitual, continuo, diario) y otras conductas de riesgo (expulsión del centro educativo, venta de drogas, robo, vandalismo, intoxicación en el centro educativo, agresión física grave, porte de armas, prácticas sexuales sin protección, viajar con un conductor que había bebido alcohol, robo a los progenitores) en formato dicotómico (sí, no).

### Análisis estadístico

Se realizó un análisis descriptivo mediante medias (x̄) y desviación típica (DT) para variables cuantitativas, y frecuencias y porcentajes para las categóricas; se aplicó el método *pairwise deletion* para los datos perdidos. Tras comprobar la aleatoriedad de las distribuciones mediante la prueba de rachas, se utilizó estadística paramétrica.

La evolución de las medidas intrasujeto en tres momentos (preintervención, posintervención y seguimiento tres meses tras la finalización) se examinó mediante modelos lineales generales de medidas repetidas con corrección de Greenhouse- Geisser cuando se vulneró el supuesto de esfericidad, y pruebas t para muestras relacionadas en los pares preintervención-posintervención, preintervención-seguimiento y posintervención-seguimiento. Las variables categóricas se contrastaron con Chi-cuadrado (χ²) y el tamaño del efecto se estimó mediante el coeficiente phi (φ), interpretado según los criterios de Cohen como pequeño (≈ 0,10), moderado (≈ 0,30) y grande (≈ 0,50). Para las comparaciones intrasujeto se calculó la d de Cohen para muestras relacionadas, interpretada como pequeña (≈ 0,20), moderada (≈ 0,50) y grande (≈ 0,80)^22^. Todos los contrastes fueron bilaterales y el nivel de significación se fijó en p <0,05. Los análisis se realizaron con el programa estadístico *Statistical Package for the Social Sciences* (SPSS), versión 28.

## RESULTADOS

### Composición de la muestra y datos sociodemográficos

Durante el periodo de estudio se produjeron 179 ingresos. De ellos se excluyeron 64 adolescentes por abandono voluntario (35,75%), cinco por expulsión del programa (2,79%), 16 por derivación a otros recursos (8,94%) y 33 por no completar las medidas de evaluación (18,44%). La muestra final estuvo integrada por 61 adolescentes (34,08%).

Predominaron los varones (n=44; 72,1%) y la edad media fue de 16,7 años (DT=2,0). La mayoría convivía con ambos progenitores (65,6%), cursaba estudios de ESO (45,9%) y no presentaba antecedentes judiciales (67,2%) ni diagnóstico psiquiátrico previo (68,9%). No se encontraron diferencias estadísticamente significativas entre hombres y mujeres para ninguna de las variables sociodemográficas analizadas (Tabla 1).

**Tabla 1:** Características sociodemográficas de la muestra de participantes en la intervención

Variable	Total	Hombre	Mujer	χ² (gl)
	(n=61)	(n=44)	(n=17)	p
	n (%)	n (%)	n (%)	
Núcleo de convivencia				0,5 (1)*
				0,49
Junto a mis padres y hermanos (ref)	40 (65,6)	30 (68,2)	10 (58,8)	
Padres separados/divorciados	9 (14,8)	7 (15,9)	2 (11,8)	
Solo con mi madre	7 (11,5)	4 (9,1)	3 (17,6)	
Junto a mi madre y su nueva pareja	3 (4,9)	1 (2,3)	2 (11,8)	
Solo con mi padre	1 (1,6)	1 (2,3)	0 (-)	
Familia adoptiva	1 (1,6)	1 (2,3)	0 (-)	
Nivel de estudios				0,9 (1)**
				0,347
ESO	28 (45,9)	21 (47,7)	7 (41,2)	
Bachiller	16 (26,2)	11 (25,0)	5 (29,4)	
FP	10 (16,4)	8 (18,2)	2 (11,8)	
Universidad (ref)	7 (11,5)	4 (9,1)	3 (17,6)	
Antecedentes judiciales	20 (32,8)	17 (38,6)	3 (17,6)	2,4 (1)
				0,117
Diagnóstico psiquiátrico previo	19 (31,1)	12 (27,3)	7 (41,2)	1,1 (1)
				0,293

gl: grados de libertad; ref: categoría de referencia para comparaciones; ESO: Educación Secundaria Obligatoria; FP: Formación Profesional.

A continuación se muestra la evolución de las variables estudiadas, desagregada por sexo.

### Variables de personalidad y competencias personales

Los hombres mostraron un cambio significativo en la variable introversión y mejoras significativas en ansiedad, autoestima, autoeficacia y planificación y toma de decisiones, con tamaños de efecto moderados a grandes, que se mantuvieron en el seguimiento. Las mujeres también mostraron cambios significativos en introversión y mejoraron en autoestima, autoeficacia, planificación y toma de decisiones, tolerancia a la frustración y resolución de conflictos (Tabla 2).

**Tabla 2:** Variables de personalidad y competencias personales. Evolución (pre-, post-, seguimiento a los tres meses) por sexo

Variable	Intervención			Comparaciones			
	Pre	Post	+3 meses	Global	Pre/Post	Pre/+3	Post/+3
	x̄ (DT)	x̄ (DT)	x̄ (DT)	p	p	p	p
					d Cohen	d Cohen	d Cohen
Hombres (n=44)							
Introversión	17,7	15,5	14,25	<0,001	0,007	<0,001	0,026
	-5,7	-4,4	-4,7		0,5	0,73	0,27
Impulsividad	12,11	11,59	11,02	0,061	0,272	0,032	0,157
	-2,8	-2,9	-3		0,18	0,36	0,19
Búsqueda de Sensaciones	19,64	20,3	20,82	0,014	0,106	0,01	0,13
	-3,8	-3,9	-3,9		0,17	0,3	0,13
Ansiedad	10,45	9,2	9,27	0,008	0,009	0,025	0,84
	-3,2	-2,3	-2,4		0,54	0,49	0,03
Autoestima	39,36	52,84	57,27	<0,001	0,008	0,001	0,161
	-31,9	-27,4	-30,2		0,49	0,59	0,15
Autoeficacia	45,27	61,7	67,16	<0,001	<0,001	<0,001	0,161
	-29,3	-25,7	-25,6		0,64	0,86	0,21
Planificación y toma de decisiones	47,39	59,02	63,18	<0,001	0,01	<0,001	0,206
	-29,2	-25,7	-24,5		0,45	0,64	0,17
Tolerancia a la frustración	42,61	48,66	52,05	0,018	0,059	0,025	0,185
	-27	-28,5	-28,9		0,21	0,33	0,12
Resolución de conflictos	48,75	55,02	56,68	0,038	0,089	0,016	0,558
	-25,5	-26,7	-26		0,23	0,31	0,06
Mujeres (n=17)							
Introversión	20,18	15,71	15,88	0,001	<0,001	0,011	0,883
	-4,9	-4,8	-4,9		0,93	0,88	0,03
Impulsividad	12,24	10,88	10,59	0,059	0,141	0,016	0,629
	-3,5	-3,2	-3,1		0,43	0,53	0,09
Búsqueda de Sensaciones	17	17,18	16,65	0,674	0,789	0,547	0,376
	-4,4	-5,1	-4,3		0,04	0,08	0,12
Ansiedad	11,94	10,88	11,35	0,579	0,297	0,563	0,655
	-4,3	-4,2	-4,4		0,25	0,13	0,11
Autoestima	34,7	55,5	59,4	<0,001	<0,001	0,005	0,501
	-27,5	-26,7	-30,1		0,78	0,82	0,13
Autoeficacia	45,6	61,8	70,9	0,005	0,069	0,007	0,065
	-32,7	-33,1	-28,8		0,49	0,88	0,32
Planificación y toma de decisiones	37,1	59,3	62,6	<0,001	<0,001	<0,001	0,531
	-23	-27,6	-25,5		0,81	1	0,13
Tolerancia a la frustración	25,6	48,2	44,4	0,001	0,002	0,002	0,548
	-24,2	-27,1	-28		0,84	0,67	0,14
Resolución de conflictos	39,8	50,2	52,4	0,042	0,022	0,04	0,694
	-26,8	-33,6	-32,7		0,31	0,39	0,07

### Sintomatología psicopatológica

Tanto hombres como mujeres mostraron mejorías estadísticamente significativas en todas las dimensiones del SCL-90-R al finalizar la intervención, manteniéndose o incrementándose en el seguimiento a los tres meses. Los tamaños de efecto fueron moderados a grandes en ambos sexos, siendo especialmente destacables las mejoras en ideación paranoide, hostilidad e Índice Global de Gravedad (GSI) (Tabla 3).

**Tabla 3:** Sintomatología psicopatológica (SCL-90-R, percentiles). Evolución pre-post-seguimiento por sexo

	Pre	Post	+3 meses	Global	Pre/Post	Pre/+3	Post/+3
	x̄ (dt)	x̄ (DT)	x̄ (DT)	p	p	p	p
					d Cohen	d Cohen	d Cohen
Hombres (*n* = 44)							
Somatización	59,66	41,82	35,93	<0,001	0,001	<0,001	0,152
	-26,5	-29,2	-28,8		0,61	0,82	0,2
Obsesión-compulsión	71,45	52,95	42,68	<0,001	<0,001	<0,001	0,006
	-23,5	-30,1	-29,7		0,61	0,97	0,35
Sensibilidad interpersonal	61,7	39,93	37,23	<0,001	<0,001	<0,001	0,478
	-30,1	-32,2	-26,3		0,68	0,93	0,1
Depresión	58,52	39,68	28,32	<0,001	0,001	<0,001	0,004
	-30,5	-29,4	27,3)		0,64	1,11	0,42
Ansiedad	61,14	44,89	37,09	<0,001	0,002	<0,001	0,048
	-28,4	-32,2	-28,9		0,5	0,83	0,27
Hostilidad	75,55	55,89	46	<0,001	<0,001	<0,001	0,014
	-22,4	-33,3	-32,2		0,59	0,92	0,31
Ansiedad fóbica	38,61	30,3	27,7	0,122	0,145	0,09	0,558
	-38,9	-30,7	-30,3		0,27	0,36	0,09
Ideación paranoide	66,25	41,66	34,8	<0,001	<0,001	<0,001	0,102
	-30,5	-32,4	-30		0,76	1,05	0,23
Psicoticismo	58,77	41,36	32,27	<0,001	0,006	<0,001	0,029
	-34,9	-33,7	-33,6		0,52	0,79	0,27
GSI	68,09	45,11	35,89	<0,001	0,004	0,004	0,052
	-26,3	-31,9	-30,2		0,72	1,07	0,31
PST	75,95	52,77	43,93	<0,001	<0,001	<0,001	0,008
	-24,1	-32,8	-33,1		0,71	0,97	0,27
PSDI	39,7	27,8	20,83	<0,001	<0,001	<0,001	0,014
	-25,6	-22,1	-16,1		0,54	1,17	0,43
Mujeres (*n* = 17)							
Somatización	62,41	29,47	27,47	<0,001	0,001	<0,001	0,544
	-26,7	-26,4	-26,2		1,25	1,33	0,08
Obsesión-compulsión	70,65	49,35	41,18	<0,001	0,018	<0,001	0,162
	-28,6	-27,9	-28,4		0,76	1,04	0,29
Sensibilidad interpersonal	73,29	48,59	43,76	<0,001	0,002	0,001	0,285
	-29,4	-32,7	-33,7		0,76	0,88	0,14
Depresión	67,94	41,59	31,53	<0,001	0,003	0,001	0,116
	-36,5	-36,6	-35,9		0,72	1,01	0,28
Ansiedad	65,35	37,59	35	<0,001	0,002	0,002	0,499
	-31,6	-33,1	-32,3		0,84	0,94	0,08
Hostilidad	80,94	52,65	46,71	<0,001	0,009	<0,001	0,408
	-24,6	-31,9	-34,9		0,89	0,98	0,17
Ansiedad fóbica	51,29	36,41	33,71	0,065	0,104	0,042	0,682
	-38,8	-40,9	-37,3		0,36	0,47	0,07
Ideación paranoide	67,65	43,59	37,35	<0,001	0,002	0,004	0,362
	-32,8	-38,5	-36,2		0,62	0,84	0,17
Psicoticismo	70,47	32,71	29,65	<0,001	<0,001	<0,001	0,661
	-34	-36,9	-36,6		1,02	1,12	0,08
GSI	73,76	40	33,47	<0,001	0,001	0,002	0,434
	-30,5	-35,9	-35,2		0,94	1,14	0,19
PST	77,18	44,76	36,41	<0,001	0,001	<0,001	0,185
	-29,2	-33,4	-35,9		0,97	1,14	0,23
PSDI	53,47	29,71	33,82	<0,001	0,001	<0,001	0,168
	-29,2	-29,9	-29,4		0,79	0,67	0,14

GSI: Índice Global de Gravedad; PST: Total de Síntomas Positivos; PSDI: Índice de Distrés de Síntomas Positivos.

### Consumo de cannabis

Al finalizar la intervención, el 68,9 % de los participantes había abandonado completamente el consumo (65,9% de los hombres y 76,5% de las mujeres). En el seguimiento a los tres meses, la abstinencia disminuyó 18,2 puntos porcentuales (al 47,7%) en hombres y 1,5 puntos en mujeres (al 75,0%). El patrón esporádico desapareció por completo tras la intervención en ambos sexos, manteniéndose en el 0% en el seguimiento. El consumo continuo mostró un aumento progresivo en los hombres (11,4% a 18,2%), mientras que en las mujeres desapareció tras la intervención. El consumo diario se redujo drásticamente en ambos sexos al finalizar el programa, si bien repuntó en hombres en el seguimiento a los tres meses (hasta el 20,5%), tendencia no observada en mujeres, donde continuó descendiendo.

No se calcularon estadísticos de contraste entre momentos dado el tamaño muestral reducido y el elevado número de casillas con frecuencia esperada inferior a 5 (Tabla 4).

Al inicio de la intervención, casi la mitad de los participantes manifestaban ser consumidores diarios; al finalizar el programa, el 68,9 % (65,9 % de los hombres y 76,5 % de las mujeres) había abandonado completamente el consumo, y la cifra ascendía al 78,7 % si se incluyen quienes lo redujeron a un patrón habitual. Además, el 70 % de quienes finalizaron el programa sin consumo y partían de la abstinencia inicial mantuvieron este estatus.

**Tabla 4:** Patrón de consumo de cannabis. Evolución pre-post-seguimiento por sexo

	Intervención		
	Pre	Post	+3 meses
	n (%)	n (%)	n (%)
Hombres (n = 44)			
Sin consumo	6 (13,6)	29 (65,9)	21 (47,7)
Esporádico	2 (4,5)	0	0
Habitual	11 (25,0)	6 (13,6)	6 (13,6)
Continuo	5 (11,4)	7 (15,9)	8 (18,2)
Diario	20 (45,5)	2 (4,5)	9 (20,5)
Mujeres (n = 16)			
Sin consumo	2 (12,5)	13 (81,3)	12 (75,0)
Esporádico	4 (25,0)	0	0
Habitual	2 (12,5)	0	2 (12,5)
Continuo	2 (12,5)	0	0
Diario	6 (37,5)	3 (18,8)	2 (12,5)

*n* = 16 en preintervención por un dato perdido.

### Evolución de otras conductas de riesgo

En los hombres se observaron reducciones estadísticamente significativas en robos en establecimientos, actos vandálicos y agresiones físicas graves entre la preintervención y la posintervención, así como en venta de drogas y porte de armas entre la preintervención y el seguimiento. En las mujeres, las frecuencias basales fueron muy reducidas, por lo que no se calcularon estadísticos de contraste (Tabla 5).

**Tabla 5:** Otras conductas de riesgo. Evolución pre-post-seguimiento por sexo.

	Intervención			Comparaciones		
Variable	Pre	Post	+3 meses	Pre/Post	Pre/+3	Post/+3
	n (%)	n (%)	n (%)	p	p	p
				φ Cohen	φ Cohen	φ Cohen
Hombres (*n* = 44)						
Expulsión del centro educativo	10 (22,7)	4 (9,1)	2 (4,5)	0,18	0,343	-
				0,21	0,14	
Vendiste drogas de consumo ilegal	19 (43,2)	7 (15,9)	8 (18,2)	0,1	0,046	-
				0,25	0,3	
Robaste en alguna tienda o supermercado	15 (34,1)	9 (20,5)	8 (18,2)	0,021	0,294	-
				0,35	0,16	
Participaste en un acto vandálico	13 (29,5)	4 (9,1)	3 (6,8)	0,036	0,147	-
				0,31	0,22	
Estuviste borracho o drogado en el centro educativo	19 (43,2)	5 (11,4)	7 (15,9)	0,078	0,1	-
				0,27	0,25	
Atacaste a alguien con la idea de herirlo gravemente	7 (15,9)	4 (9,1)	2 (4,5)	0,001	0,18	-
				0,51	0,2	
Llevaste un arma	7 (15,9)	2 (4,5)	3 (6,8)	0,181	0,013	-
				0,2	0,37	
No usaste preservativo u otro anticonceptivo en alguna actividad sexual	13 (30,2)	10 (22,7)	5 (11,4)	0,121	0,584	-
				0,24	0,08	
Viajaste con un conductor que había bebido alcohol	16 (36,4)	10 (22,7)	12 (27,3)	0,254	0,065	-
				0,18	0,28	
Robaste joyas u oro a tus padres para venderlas	2 (4,5)	1 (2,3)	0	-	-	-
Mujeres (*n* = 17)						
Expulsión del centro educativo	1 (6,3)	1 (6,3)	0	-	-	-
Vendiste drogas de consumo ilegal	2 (12,5)	0	0	-	-	-
Robaste en alguna tienda o supermercado	3 (18,8)	3 (18,8)	2 (12,5)	-	-	-
Participaste en un acto vandálico	0	0	0	-	-	-
Estuviste borracha o drogada en el centro educativo	4 (25,0)	1 (6,3)	1 (6,3)	-	-	-
Atacaste a alguien con la idea de herirlo gravemente	1 (6,3)	0	0	-	-	-
Llevaste un arma	0	0	1 (6,3)	-	-	-
No usaste preservativo u otro anticonceptivo en alguna actividad sexual	4 (25,0)	2 (12,5)	2 (12,5)	-	-	-
Viajaste con un conductor que había bebido alcohol	4 (25,0)	4 (25,0)	2 (12,5)	-	-	-
Robaste joyas u oro a tus padres para venderlas	4 (25,0)	0	0	-	-	-

-: un porcentaje >20% de casillas con recuento esperado <5 impidió realizar comparaciones.

## DISCUSIÓN

Los resultados de este estudio apoyan la eficacia del Programa Suspertu en variables de personalidad, sintomatología psicopatológica, consumo de cannabis y otras conductas de riesgo en adolescentes de la Comunidad Foral de Navarra. Las mejoras se mantienen, e incluso aumentan, a los tres meses de seguimiento, en línea con lo descrito en programas de prevención indicada de orientación cognitivo-conductual y trabajo familiar^8^^,^^9^^,^^10^^,^^23^.

En el análisis de la evolución de las características de personalidad se observan cambios significativos en la introversión y mejoras en los niveles de ansiedad desde el inicio hasta la finalización de la intervención. Clínicamente podría atribuirse esta mejoría a que el espacio terapéutico facilitaría la verbalización de las dificultades y reduciría la sensación de aislamiento, aunque el diseño del estudio no permite contrastar esta hipótesis. En el seguimiento a los tres meses se evidencia un aumento significativo en la búsqueda de sensaciones, aspecto consistentemente relacionado con comportamientos de riesgo, especialmente el consumo de drogas^6^^,^^7^^,^^12^^,^^24^^,^^25^, que apoyaría la hipótesis de que la búsqueda de sensaciones es un rasgo inherente a la adolescencia, parcialmente adaptativo y difícilmente modificable^3^^,^^4^^,^^24^. Asimismo, los cambios en las dinámicas familiares y en el estilo de vida, que probablemente exigen un mayor autocontrol, podrían acentuar la expresión de esta dimensión.

Las variables personales (autoestima, autoeficacia, planificación y toma de decisiones, tolerancia a la frustración) mejoraron con tamaños de efecto moderados a grandes, y estos cambios se mantuvieron o incluso se incrementaron en el seguimiento, especialmente en autoeficacia percibida. Este patrón es coherente con los contenidos modulares trabajados en el programa, dirigidos específicamente a estas áreas, y converge con la evidencia previa que identifica estas variables como mediadores relevantes en intervenciones psicoeducativas con adolescentes^5^^,^^8^^,^^9^.

Las mujeres, a pesar de presentar puntuaciones basales más afectadas en varias dimensiones, mostraron una magnitud de cambio en general algo menor que la observada en los hombres, lo que subraya la importancia de considerar enfoques diferenciados según el sexo en futuras intervenciones, en línea con la evidencia disponible sobre diferencias de género en programas de prevención y tratamiento^10^^,^^26^.

Los resultados en sintomatología psicopatológica indican una disminución generalizada de la sintomatología, con estabilización o consolidación de las mejoras en el seguimiento a los tres meses, lo que sugiere una consistencia de los cambios en el corto plazo^8^.

El cannabis era la sustancia más consumida al ingreso, y la intervención se asoció a una reducción marcada del consumo, que se mantuvo mayoritariamente en el seguimiento. Estos resultados son coherentes con los objetivos centrales del programa y con los obtenidos en intervenciones similares en el contexto español^9^^,^^10^. Cabría plantearse si la reducción del consumo está detrás de buena parte de las mejoras observadas en otras áreas, hipótesis que el diseño no permite confirmar pero que se apoya en el solapamiento descrito entre cannabis y problemas psicopatológicos y conductuales en adolescentes^6^^,^^7^^,^^25^. En el seguimiento a los tres meses se observa un ligero repunte en el consumo de cannabis, especialmente en los hombres. Este patrón es habitual en los programas de prevención indicada^27^ y subraya la necesidad de mantener componentes de seguimiento más allá del cierre de la intervención^28^.

Al final de la intervención se observó un descenso generalizado en otras conductas de riesgo, que en su mayor parte se mantiene en el seguimiento. La frecuente coexistencia entre el consumo problemático de cannabis y este tipo de conductas en adolescentes^6^^,^^25^ sugiere que la reducción del consumo podría haber contribuido a estos cambios, aunque, como se ha señalado, el diseño no permite establecer relaciones causales.

El presente estudio presenta varias limitaciones que deben tenerse en cuenta al interpretar los resultados. En primer lugar, la dificultad para obtener datos de los participantes con salida negativa del programa (por abandono o incumplimiento de condiciones) ha impedido tanto el análisis comparativo entre salidas positivas y negativas como el estudio de las características iniciales de quienes no completaron la intervención, lo que limita la posibilidad de determinar en qué medida los participantes que completaron el programa son representativos del conjunto de adolescentes que ingresan en él. Esta es una limitación reconocida como habitual en este tipo de investigaciones, con implicaciones relevantes para la validez externa de los resultados^29^. Cabe señalar, además, que los participantes que completaron el programa y las evaluaciones podrían representar los casos con mejor evolución y mayor adherencia, lo que introduciría un sesgo de selección que tendería a sobreestimar la eficacia de la intervención. En segundo lugar, la notable disparidad entre hombres y mujeres en la muestra (con 44 hombres frente a tan sólo 17 mujeres), reflejo de los patrones de atención propios del programa, condiciona la generalización de los resultados, una limitación recurrente en la investigación sobre intervenciones en jóvenes^26^. En tercer lugar, el periodo de seguimiento de tres meses limita la posibilidad de evaluar la durabilidad de los cambios a más largo plazo. Por último, al tratarse de medidas autoinformadas, no puede descartarse la influencia del sesgo de deseabilidad social en las respuestas de los participantes^30^. Finalmente, la ausencia de un grupo de comparación constituye una limitación inherente al diseño cuasi-experimental, que limita la posibilidad de atribuir los cambios observados exclusivamente a la intervención y representa una línea de mejora metodológica para futuros estudios.

De cara a futuras investigaciones, sería recomendable incorporar el análisis de los participantes con salida negativa, comparando sus características al inicio, momento del ingreso, perfil de consumo, variables de personalidad y contexto familiar, con las de quienes completaron la intervención. Ello permitiría identificar predictores tempranos de abandono, orientar estrategias de retención más eficaces y avanzar hacia intervenciones más ajustadas a los perfiles de mayor riesgo. Asimismo, el cruce de los datos de los adolescentes con los de sus familias ofrecería una visión más integral de los efectos del programa. Finalmente, la realización de seguimientos a más largo plazo y el análisis de subgrupos específicos -como adolescentes con diagnóstico de Trastorno por Déficit de Atención e Hiperactividad (TDAH) o en situación de adopción- constituirían líneas de trabajo de especial interés para profundizar en la eficacia del programa. Del mismo modo, los resultados diferenciales observados entre hombres y mujeres apuntan a la necesidad de explorar adaptaciones específicas de la intervención en función del sexo, tanto en los contenidos trabajados con los adolescentes como en el enfoque de la intervención familiar, con el fin de optimizar la respuesta terapéutica en cada grupo.

En conjunto, los resultados de este estudio respaldan la utilidad del Programa Suspertu como intervención de prevención indicada, observándose mejoras en variables de personalidad, sintomatología psicopatológica, consumo de cannabis y otras conductas de riesgo, que se mantienen o incluso se consolidan a los tres meses de seguimiento. No obstante, las diferencias observadas entre hombres y mujeres, así como el repunte del consumo de cannabis en el seguimiento, sugieren la necesidad de reforzar los componentes de mantenimiento tras la intervención y de avanzar hacia enfoques diferenciados según el sexo.

## Data Availability

Los datos que respaldan los resultados de este estudio están disponibles a petición razonada dirigida al autor de correspondencia (suspertu@proyectohombrenavarra.org), sujeta a las restricciones éticas y legales aplicables a datos clínicos de menores de edad.
